# Detectable Virological Load and Associated Factors among People Living with HIV on Antiretroviral Treatment: A Retrospective Study

**DOI:** 10.3390/pathogens13050359

**Published:** 2024-04-27

**Authors:** Pierpaolo Congedo, Raffaella Sedile, Marcello Guido, Federico Banchelli, Antonella Zizza

**Affiliations:** 1 Infectious Diseases Unit, Vito Fazzi Hospital, 73100 Lecce, Italy; congedopierpaolo1961@gmail.com; 2 Institute of Clinical Physiology, National Research Council, 73100 Lecce, Italy; raffaella.sedile@cnr.it (R.S.); antonella.zizza@cnr.it (A.Z.); 3 Laboratory of Hygiene, Department of Biological and Environmental Sciences and Technologies, University of Salento, 73100 Lecce, Italy; 4 Department of Medical and Surgical Sciences, University of Modena and Reggio Emilia, 41100 Modena, Italy; federico.banchelli@gmail.com

**Keywords:** antiretroviral therapy, detectable viral load, associated factors, HIV/AIDS

## Abstract

The complete and prolonged suppression of viral load is the primary objective of HAART in people living with HIV. Some people may experience therapeutic failure, while others may achieve virological suppression but are unable to maintain it, developing persistent or single detection of low-level viremia. This study aims to evaluate the determinants of a detectable viral load among patients on HAART to identify and address them promptly. In this retrospective study, all patients referring to the Infectious Disease Operative Unit of the Vito Fazzi Hospital in Lecce, Puglia, older than 18 years, receiving HAART for at least 12 months as of 30 June 2022, were included. For each patient, demographic characteristics such as age, sex, educational level, stable relationship, cohabitation, employment status, and information relating to habits and lifestyles such as physical activity, use of drugs, and substances or supplements for sport, abuse of alcohol, and smoking were collected. Degree of comorbidity was quantified according to the Charlson Comorbidity Index, and the presence of obesity and the COVID-19 infection was also considered. Univariable and multivariable logistic regression models were used to assess the association between patients’ characteristics and the outcome. In the multivariable logistic regression model, the odds were lower for the duration of therapy (OR: 0.96; *p* = 0.0397), prescriber’s perception of adherence to therapy (OR: 0.50; *p* < 0.0001), and Nadir CD4+ T-cell count (OR: 0.85; *p* = 0.0329), and higher for the presence of AIDS (OR: 1.89; *p* = 0.0423) and COVID-19 (OR: 2.31; *p* = 0.0182). Our findings support the early initiation of HAART to achieve virological suppression. Additionally, measures to improve adherence to therapy should be adopted to ensure better outcomes for patients.

## 1. Introduction

The use of highly active antiretroviral therapy (HAART) represented a fundamental turning point in the management of HIV/AIDS, so much so that it is no longer included among the top ten leading causes of death in the world [1]. The reduction in the number of deaths per year from 1.0 million in 2016 to 630,000 in 2022 [2], as well as in the number of new diagnoses, represents encouraging results for achieving the 95-95-95 targets set by UNAIDS to eradicate HIV/AIDS, according to which, by 2025, 95% of all people living with HIV (PLWH) will know their status, 95% of people diagnosed with HIV infection will receive antiretroviral therapy, and 95% of all people on treatment will achieve suppression of viral load (VL) [3].

However, to date, it is estimated that there are approximately 5.5 million people in the world who still do not know they are HIV positive, which leads to a high number of late diagnoses of HIV [2]. In Italy, as in the rest of the world, the incidence of AIDS has reduced in recent years while the number of late diagnoses, i.e., subjects who discovered their HIV-positive status a few months before the diagnosis of AIDS, has increased [4]. This may also be due to poor knowledge and awareness of the risk for contracting the infection [5].

Although there is no cure for PLWH and lifelong treatment is required, HAART has revolutionized the course of the HIV infection by restoring the immune system and keeping VL at undetectable levels, thereby transforming the HIV infection into a chronic, non-fatal condition. The complete and prolonged suppression of detected VL is the primary objective of therapy [6], which allows us to control viral replication but not its complete eradication, so the occurrence of even temporary therapeutic failures constitutes an obstacle to achieving control of the HIV/AIDS epidemic.

People who are receiving HAART and who achieve and maintain an undetectable VL do not transmit HIV to their sexual partners [7]. The key to achieving this goal is regular monitoring of the treatment outcome via VL testing [8]. It is recommended that PLWH undergo VL testing when they start treatment, every 2–8 weeks until virological suppression (VS) is achieved, every 3–4 months in virologically suppressed patients, and every 6 months in patients with VS for at least 2 years [9].

Some authors have shown that the nadir of the HIV RNA copy number in plasma following HAART is a predictor of the duration of the virological effect (the lower this level, the longer the duration of the effect) [10].

The suppression of VL below the assay quantification cut-off is widely recognized as an indicator of HAART success, although definitions of VS and virological failure can vary considerably among different regions and countries, depending on medical and socioeconomic factors [9,11,12,13]. During therapy, some patients may experience therapeutic failure [14], while others may achieve VS but may not be able to maintain it, leading to a virological rebound, which is a prerequisite for virological failure [15] and/or resistance as well as vulnerability to the disease [16].

During treatment, some patients may develop persistent low-level viremia (pLLV), which is defined as two or more consecutive episodes of a virological rebound with VL above the lower detection limit of the test (<20 or <50 copies/mL) but below the threshold that defines virological failure (>200 or >1000 copies/mL depending on the reference guidelines). Other patients may experience a viral blip, which is a single detection of low-level viremia [11,12,13].

Recent research has revealed that patients with low-level viremia (51–199 copies per ml) are at risk of subsequent VL of at least 200 copies per mL [17]. Low levels of viremia can be linked to the latent viral reservoir and depend on the time elapsed since the start of therapy, Zenith VL, and Nadir CD4+ T-cells [18].

In a recent review, insufficient adherence to treatment, the presence of co-infections, low CD4+ T-cell count, and high VL at the initiation of HAART were reported as the main factors contributing to virological failure [14]. Poor adherence could allow for periods of viral replication, leading to the development of drug resistance and resulting in limited treatment efficacy [19]. Furthermore, social and economic factors, such as age, marital status, educational level, coming out, poverty, unemployment, and the lack of food can influence treatment adherence [20,21].

Other factors can contribute to the ineffectiveness of treatment. The duration of therapy and changes in the therapeutic regimen can increase the likelihood of therapeutic failure [22,23]. Furthermore, alcohol abuse appears to increase the number of doses of medications not taken and interruptions in treatment, subsequently leading to therapeutic failure [24].

Therefore, this study aims to evaluate the determinants of a detectable virological load among patients undergoing HAART therapy to allow us to promptly identify and control these factors.

## 2. Materials and Methods

### 2.1. Study Design and Population

This study was conducted at the Infectious Disease Operative Unit (OU) of the Vito Fazzi Hospital in Lecce, Puglia. All patients older than 18 years who were receiving HAART for at least 12 months as of 30 June 2022 were included in this retrospective observational study. Data were collected from follow-up visits during the first half of 2021 (1 January–30 June) and the first six months of 2022 (1 January–30 June).

This study was performed in accordance with the Declaration of Helsinki and approved by the Ethics Committee of the Health Local Unit of Lecce (Report n.103 of 2 February 2023).

For the present study, only patients treated at the Infectious Disease OU of the Vito Fazzi Hospital since the first therapy, for whom all the required information was available, were considered eligible, out of a total of 550 patients followed. Demographic characteristics such as age, sex, educational level, stable relationship, cohabitation, employment status, and information relating to habits and lifestyles such as physical activity, use of drugs, and substances or supplements for sport, abuse of alcohol, and smoking were collected for each patient. The degree of comorbidity was quantified according to the Charlson Comorbidity Index (2011 updated version) [25] excluding AIDS, and the presence of obesity and the COVID-19 infection in the period between 2021 and 2022 was also considered.

We used a modified version of the Charlson index, considering the presence of AIDS as a separate variable, in order to better evaluate its possible effect on the response to therapy. We considered the condition of AIDS based on the appearance of AIDS-defining opportunistic diseases.

Furthermore, clinical data of patients such as time from HIV diagnosis to start of HAART, the overall duration of therapy, disclosure of HIV Status, no. of drugs classes, switch to a single-tablet regimen, time since the start of current therapeutic plan, change of therapeutic plan due to failure or voluntary suspension or for optimization, number of daily pills for other pathologies except HAART, use of gastroprotective drugs, and laboratory data (Nadir CD4+ T-cells; Zenith VL) were retrospectively collected from patients’ medical records.

Adherence to therapy was quantified on a scale of 1 to 10 based on the perception of the prescriber, an HIV specialist doctor who had over thirty years of experience and followed the patients included in this study from the beginning of their HIV diagnosis. The score was calculated on based of objective data, such as regularity in medical checks and punctuality in collecting antivirals from the hospital pharmacy, the onset of comorbidities, and on the doctor’s perception of the regularity in taking the therapy and lifestyle habits (use of narcotic substances, psychotropic drugs, alcohol abuse, significant changes in daily life), as reported by the patient during the interviews.

Patients were classified as having detectable or undetectable virological load based on HIV RNA levels determined using the cobas^®^ HIV-1 quantitative nucleic acid test via cobas^®^ 6800/8800 Systems (Roche Diagnostics), with limits of quantification between 20 and 10,000,000 copies/mL.

By the term detectable virological load (D-VL), we mean patients with a viral blip, pLLV, or virological failure, with the viral blip representing a nonconsecutive episode of VL > 20 copies/mL (corresponding to the minimum level of copies detectable by the laboratory test); for pLLV, at least two consecutive measurements with VL between 20 and 200 copies/mL; and for virological failure, VL with consistently >200 copies/mL.

Patients with VL of less than 20 copies/mL were classified as undetectable virological load (U-VL).

### 2.2. Statistical Analysis

Descriptive statistics were reported as mean and standard deviation for numerical variables and as numbers and percentages for categorical variables. To assess the association between patient characteristics and the D-VL binary outcome in a primary analysis, univariable and multivariable logistic regression models were used. The final logistic model was determined by a stepwise backward variable selection process, retaining the variables that improved the Akaike information criterion. As an additional analysis, a multivariable multinomial logistic model was used to assess the influence of these variables on the occurrence of a viral blip and on the occurrence of pLLV or virological failure. Results of all analyses were reported as odds ratios (ORs). Uncertainty was reported with 95% confidence intervals (95% CIs). Given the retrospective exploratory design, the sample size was not formally calculated but was determined by the number of eligible individuals within the period of interest. Sample size was judged to be adequate based on the ratio of the number of events to the number of explanatory variables. Possible findings of interest were the adjusted associations with *p* < 0.05 or, although with a lower level of certainty, *p* < 0.1. Analyses were carried out with R 3.4.3 statistical software (The R Foundation for Statistical Computing, Wien, Austria).

## 3. Results

This study included 338 people living with HIV-1 (260 men and 78 women, age ranging from 25 to 77 years). The patients had a mean age of 47.69 ± 11.24 years.

Based on their responses to HAART therapy, the patients were divided into two groups: U-VL (163 patients) and D-VL (175 patients) (Table 1). The D-VL group included 14 virological failures, 70 pLLV, and 91 viral blips.

The U-VL group had an average age of 47.20 ± 11.05 years, while the group of D-VL had an average age of 48.14 ± 11.43 years.

Table 1 shows the individual and clinical characteristics of the study groups.

The univariate analysis revealed that education stage > 10 years (OR: 0.63; *p* = 0.0352), use of single-tablet regimen for HAART (OR: 0.51; *p* = 0.0031), time since the start of the current therapeutic plan (OR: 0.93; *p* = 0.0460), prescriber’s perception of adherence to therapy (OR: 0.52; *p* < 0.0001), and Nadir CD4+ T-cell count (OR: 0.76; *p* < 0.0001) were correlated with a reduction in the risk of D-VL, while alcohol abuse (OR: 4.88; *p* = 0.0428), smoking more than 20 cigarettes a day (OR: 1.91; *p* = 0.0206), presence of AIDS (OR: 2.60; *p* = 0.0002), change of therapeutic plan due to failure or voluntary suspension (OR: 2.55; *p* = 0.0076), and no. of daily tablets except HAART (OR: 1.15; *p* = 0.0301) were linked with an increased risk of D-VL.

In the multivariable analysis, the presence of COVID-19 (OR: 2.23; *p* = 0.0339) and AIDS (OR: 2.04; *p* = 0.0354) were associated with an increased risk of incomplete response, whereas prescriber’s perception of adherence to therapy (OR: 0.50; *p* < 0.0001) reduced the risk.

The use of the single-tablet regimen (OR: 0.58; *p* = 0.0765) and Nadir CD4+ T-cell count levels (OR: 0.85; *p* = 0.0617) highlight a possible relationship with the reduction in incomplete response risk.

In the multivariable backward stepwise logistic regression model retaining the variables that improved the Akaike information criterion, the odds were lower for the duration of therapy (OR: 0.96; *p* = 0.0397), prescriber’s perception of adherence to therapy (OR: 0.50; *p* < 0.0001), and Nadir CD4+ T-cell count (OR: 0.85; *p* = 0.0329), and higher for the presence of AIDS (OR: 1.89; *p*= 0.0423) and COVID-19 (OR: 2.31; *p* = 0.0182).

Moreover, the single-tablet regimen (OR: 0.61; *p* = 0.0698) may have a possible relationship with a reduction in the risk of an incomplete response (Table 2).

The findings of the additional multinomial logistic regression model that was conducted to evaluate the influence of variables on the occurrence of BV are quite in line with the main analysis. Particularly, odds were lower for the prescriber’s perception of therapy adherence (OR: 0.59; *p* < 0.0001) and Nadir CD4+ T-cell count (OR: 0.84; *p* = 0. 0584), and higher for the presence of AIDS (OR: 1.97; *p* = 0.0515) and COVID-19 (OR: 2.68; *p* = 0.0099) (Table 3).

The additional multinomial logistic regression model that was conducted to assess the influence of the variables on the occurrence of pLLV and virological failure showed lower odds for the prescriber’s perception of adherence to therapy (OR: 0.42; *p* < 0.0001) and for the duration of therapy (OR: 0.95; *p* = 0.0534) (Table 4).

## 4. Discussion

The introduction of HAART has been one of the most significant achievements in controlling the spread of HIV due to its effect on viral suppression [13]. However, despite the efficiency of the treatment, some patients still experience virological failure or suboptimal response in reducing VL to undetectable levels [26,27,28,29]. The suboptimal response is particularly studied in high-income countries where it has become evident with the introduction of tests that can quantify HIV viral loads below 50 copies/mL [18,30,31].

Among the risk factors associated with virological failure or incomplete virological response, socio-demographic (age, sex, education level, marital status) and clinical factors (adherence to therapy, duration of therapy, low CD4+ T-cell count, and high VL at the initiation of HAART) have been identified [14,22].

A study conducted in Europe has suggested that high baseline plasma levels of HIV-1 RNA and a low CD4+ T-cell count were associated with lower rates of virological suppression and higher rates of viral blips and low-level viremia [18].

Another cohort study conducted by Alvares et al., in 2023, found similar results [32].

In addition, a recent study on a large Italian cohort has shown that individuals with a lower Nadir CD4+ T-cell count, a longer history of HIV infection, and a higher Zenith viremia pose a higher risk of low-level viremia [33].

Our study showed a correlation between the presence of AIDS and COVID-19 and an increased risk of D-VL, and among high levels of Nadir CD4+ T-cells, longer duration of therapy, higher prescriber’s perception of adherence, and a reduced risk of D-VL.

The CD4+ T-cell count is the cornerstone of immunity that helps the human body in preventing disease and HIV replication [34]. Evidence has indicated that a lower CD4+ T-cell count is related to a high VL [33,35].

Immunocompromised patients are more vulnerable to several opportunistic infections, which can lead to viral replication and a higher risk of drug resistance [35].

When the patient’s immune status is compromised, the rate of viral replication increases, which is in agreement with our study’s findings for patients with a complete AIDS state prior to the observation period, which is characterized by the development of one or more opportunistic diseases regardless of the CD4+ T-cell count.

Therefore, current guidelines recommend early diagnosis and timely initiation of HAART when the immune system is not yet severely compromised [36]. Delaying HIV diagnosis and initiation of therapy can lead to a poor response to HAART [37].

In addition, we found that a longer duration of therapy is associated with a better therapeutic response. Some authors highlighted variations in the trend of the immuno-virological response in relation to the duration of therapy. They found that concordant positive responders comprised 60.5% of the subjects at 6 months of treatment, and this value was increased to 62.8% at 12 months, whereas the percentage of concordant negative responders decreased from 9.3% to 8.1% [38]. Similarly, in a recent study, the duration of therapy ≤ 6 years was associated with immunological failure, and it was two times more likely to cause failure than a duration of follow-up on HAART of >6 years [22].

However, the relationship between the duration of therapy and the therapeutic response shows conflicting results. In a recent meta-analysis, three studies demonstrated that a longer duration of therapy increased the odds of virological failure by 1.81 times compared to a shorter duration [39]. The discrepancy between these results may be due to the lack of evaluation of HAART drug-resistant virus strains and their prevalence rate, which also represents a limitation of our study. It is possible, however, that a longer duration of therapy increases the likelihood of developing side effects from drug, discontinuing therapy, or resulting in poor adherence [39,40].

Our analysis highlights that patients who are perceived to be more adherent to therapy have a lower risk of D-VL. The relationship between treatment adherence and effectiveness of HAART is well documented in the literature [41]; high levels of adherence permit to achieve viral suppression and prevent disease progression [42].

Currently, there is no gold standard for evaluating patient adherence to HAART therapy. Several methods have been used in clinical trials, including patient self-assessment, data provided by the pharmacy, electronic system for monitoring therapeutic events, and blood monitoring of drugs. However, each of these methods may present critical issues [43,44,45]. Among the different methodologies for measuring adherence to therapy, we used an approach based on a doctor’s perception during follow-up visits.

This method introduces subjectivity that, although not comparable to other independent measures of adherence, reflects real-world experience and may therefore be a useful approach for the HIV care community.

A recent meta-analysis of 38 different studies showed that an assessment of medication adherence by a physician was more likely to predict virological failure than patient self-assessment or pharmacy data [46].

HAART regimens differ in dosing complexity, toxicity, and tolerability, which can affect treatment adherence and its outcome [47]. Among the factors that contribute to this, the number of tablets administered daily is one of the main obstacles that requires a high level of commitment and adaptation from the patient [48]; therefore, most international guidelines recommend reducing the burden of having to take many pills to prevent non-adherence to treatment [9,12,13].

Simplifying treatment through a single-tablet regimen has been linked to better adherence [49] and improved quality of life [50].

Although with a higher level of uncertainty, our findings suggest a possible reduction in the risk of D-VL with the use of a single-tablet regimen. Previous studies have showed that patients treated with a single-tablet regimen were more adherent than those treated with multi-tablet regimens and had a higher rate of VL suppression at 48 weeks [51].

Adherence to medication may also be influenced by other factors, such as the relationship with the doctor, ease of access to healthcare services and information, clinical and laboratory follow-up, and treatment success [43]. Therefore, monitoring patients’ adherence to therapy and taking appropriate measures to improve it are key actions to ensure an adequate response to treatment.

The correlation between the presence of COVID-19 and D-VL also emerged from our multivariable analysis. Subjects with comorbidities tend to be more exposed to contracting SARS-CoV-2 and experiencing a more severe course of the disease [52]; therefore, it is more likely that the correlation between the presence of COVID-19 and the risk of D-VL depends on the reduced T-cell response in patients with detectable viremia [53,54]. We cannot exclude the fact that this result could constitute a confounding factor, as we did not compare pre- and post-pandemic data. Limited access to healthcare services due to lockdown policies has certainly led to discontinuity in access to care, even for PLWH. However, recent studies suggest that missed visits and reduced VL monitoring during the COVID-19 pandemic were not associated with worse virological outcomes in most cases [55,56].

Our study was carried out at the UO of Infectious Diseases of the “Vito Fazzi” Hospital, which treats the majority of PLWH in the province of Lecce. Moreover, to our knowledge, this is the first study conducted in this area to investigate factors associated with detectable VL in PLWH treated with HAART. Multiple consecutive VL measurements throughout the observation period were considered to avoid the misclassification of patients with detectable VL.

However, this is a study conducted in a single hospital in which the observation period did not exceed 12 months; for this reason, some potential unmeasured confounding factors could also lead to differences between groups. Moreover, the sample size is not high compared to the number of explanatory variables that were assessed. This study did not test for HIV drug resistance, genetic polymorphisms, or miRNA analysis, all of which can influence the therapeutic response [28,57]. Therefore, further studies are needed to investigate genetic factors as possible causes of suboptimal or nonpersistent responses to therapy. Furthermore, the use of backward selection methods may have introduced a risk of overfitting in the statistical models.

Multicenter studies with larger sample sizes are also necessary to examine the different subgroups defined in this study in more detail.

Despite these limitations, this study provides information that can be useful for HAART treatment programs.

## 5. Conclusions

Identifying factors associated with detectable VL allows for a more appropriate use of therapy as well as for avoiding side effects of HAART and preventing drug resistance against new viral strains.

Furthermore, identifying these factors can reduce the economic burden of therapeutic failure and allow us to implement preventive strategies to reduce the failure rate.

This study suggests that the presence of AIDS and COVID-19 are factors associated with the risk of D-VL, while the duration of HAART and prescriber’s perception of adherence to therapy and Nadir CD4+ T-cell levels are associated with a lower risk of D-VL.

Our findings support the early initiation of HAART to achieve the goal of virological suppression. Furthermore, measures to optimize adherence to therapy should be adopted.

## Figures and Tables

**Table 1:** Patient baseline characteristics.

Variables	Undetectable Virological Load (n = 163)	Detectable Virological Load (n = 175)	All Patients (n = 338)
Age, years (mean ± DS)	47.20 ± 11.05	48.14 ± 11.43	47.69 ± 11.24
Sex, female (n, %)	43 (26%)	35 (20%)	78 (23%)
Time from diagnosis to start of HAART, years (mean ± SD)	1.90 ± 3.63	1.97 ± 4.40	1.93 ± 4.04
Duration of therapy, years (mean ± SD)	10.40 ± 7.93	9.67 ± 7.58	10.02 ± 7.75
Disclosure of HIV status (n, %)	89 (55%)	100 (57%)	189 (56%)
Education stage > 10 years (n, %)	96 (59%)	83 (47%)	179 (53%)
Stable relationship (n, %)	86 (53%)	93 (53%)	179 (53%)
Lives alone (n, %)	35 (21%)	33(19%)	68 (20%)
Employed (n, %)	110 (67%)	118 (67%)	228 (67%)
Occasional physical activity (n, %)	58 (36%)	49 (28%)	107 (32%)
Habitual physical activity (n, %)	21 (13%)	19 (11%)	40 (12%)
Use of substances or supplements for sport (n, %)	16 (10%)	10 (6%)	26 (7.7%)
Drug use (n, %)	9 (6%)	109 (11%)	28 (8.3%)
Alcohol abuse (n, %)	2 (1%)	10 (6%)	12 (4%)
Smoking up to 20 cigarettes a day (n, %)	47 (29%)	50 (29%)	97 (29%)
Smoking more than 20 cigarettes a day (n, %)	30 (18%)	50 (29%)	80 (24%)
AIDS (n, %)	29 (18%)	63 (36%)	92 (27%)
No. of drug classes ^#^ since start of therapy (mean ± SD)	2.67 ± 0.80	2.80 ± 0.87	2.74 ± 0.84
Single-tablet regimen for HAART (n, %)	115 (71%)	96 (55%)	211 (62%)
Time since start of current TP, years (mean ± SD)	4.23 ± 2.97	3.60 ± 2.75	3.90 ± 2.87
Change of TP due to failure or voluntary suspension (n, %)	17 (10%)	38 (22%)	55 (16%)
Change of TP for optimization (n, %)	90 (55%)	88 (50%)	178 (53%)
Charlson Comorbidity Score, median (IQR) *	0.45 (1.19)	0.58 (1.13)	0.52 (1.16)
COVID-19 (n, %)	19 (12%)	32 (18%)	51 (15%)
No. of daily tablets except HAART (mean ± SD)	1.07 ± 1.53	1.52 ± 2.10	1.30 ± 1.86
Gastroprotective drugs (n, %)	46 (28%)	66 (38%)	112 (33%)
Prescriber’s perception of adherence to therapy ° (mean ± SD)	9.08 ± 1.01	8.03 ± 1.54	8.54 ± 1.41
Nadir CD4+ T-cell count, cells/mL (mean ± SD)	296.99 ± 208.70	201.63 ± 165.94	247.62 ± 193.47
Zenith viral load, log10copies (mean ± SD)	421,135.3 ± 1,317,820.7	447,472.5 ± 842,150.7	434,771.4 ± 1,095,986.7
Obesity (n, %)	20 (12%)	29 (17%)	49 (14%)

SD, standard deviation; TP, therapeutic plan; ^#^ NRTI, NNRTI, PI, II, and FI. * Charlson Comorbidity Score (2011 updated version—excluding AIDS); ° scale from 0 to 10.

**Table 2:** Univariate and multivariable logistic regression with backward stepwise selection predicting a detectable virological load.

Variables	Univariate			Multivariable			Multivariable Backward Stepwise		
	OR	95% CI	*p*-Value	OR	95% CI	*p*-Value	OR	95% CI	*p*-Value
Age, years	1.01	0.99–1.03	0.4445	1.02	0.99–1.05	0.2107	1.02	0.99–1.05	0.1256
Sex, female	0.70	0.42–1.16	0.1652	0.80	0.40–1.61	0.5352			
Time from diagnosis to start of HAART, years	1.00	0.95–1.06	0.8844	0.99	0.93–1.07	0.8652			
Duration of therapy, years	0.99	0.96–1.02	0.3826	0.96	0.91–1.01	0.1244	0.96	0.92–1.00	0.0397
Disclosure of HIV status	1.11	0.72–1.70	0.6382	1.25	0.73–2.14	0.4202			
Education stage >10 years	0.63	0.41–0.97	0.0352	1.04	0.59–1.83	0.9041			
Stable relationship	1.02	0.66–1.56	0.9439	1.08	0.62–1.90	0.7839			
Lives alone	0.85	0.50–1.45	0.5492	0.75	0.36–1.56	0.4441			
Employed	1.00	0.63–1.57	0.9912	1.32	0.71–2.46	0.3767			
Occasional physical activity	0.66	0.41–1.07	0.0906	0.99	0.53–1.87	0.9758			
Habitual physical activity	0.71	0.36–1.41	0.3264	2.33	0.80–6.81	0.1227			
Use of substances or supplements for sport	0.56	0.25–1.27	0.1621	0.41	0.12–1.45	0.1666			
Drug use	2.08	0.91–4.75	0.0806	1.48	0.52–4.22	0.4682			
Alcohol abuse	4.88	1.05–22.61	0.0428	2.29	0.39–13.57	0.3622			
Smoking up to 20 cigarettes a day	1.22	0.74–2.02	0.4400	0.84	0.45–1.57	0.5809			
Smoking more than 20 cigarettes a day	1.91	1.10–3.31	0.0206	1.23	0.60–2.52	0.5704			
AIDS	2.60	1.57–4.31	0.0002	2.04	1.05–3.96	0.0354	1.89	1.02–3.50	0.0423
No. of drug classes ^#^ since start of therapy	1.21	0.93–1.56	0.1513	0.86	0.55–1.35	0.5233			
Single-tablet regimen for HAART	0.51	0.32–0.80	0.0031	0.58	0.31–1.06	0.0765	0.61	0.35–1.04	0.0698
Time since start of current TP, years	0.93	0.86–1.00	0.0460	0.94	0.85–1.04	0.2287	0.93	0.85–1.02	0.1090
Change of TP due to failure or voluntary suspension	2.55	1.28–5.09	0.0076	1.78	0.67–4.75	0.2499			
Change of TP for optimization	1.12	0.69–1.81	0.6523	1.38	0.66–2.87	0.3953			
Charlson Comorbidity Score *	1.10	0.91–1.32	0.3302	0.96	0.74–1.23	0.7251			
COVID-19	1.70	0.92–3.13	0.0912	2.23	1.06–4.68	0.0339	2.31	1.15–4.63	0.0182
No. of daily tablets except HAART	1.15	1.01–1.29	0.0301	1.09	0.92–1.28	0.3215			
Gastroprotective drugs	1.54	0.97–2.43	0.0646	0.87	0.46–1.64	0.6659			
Prescriber’s perception of adherence °	0.52	0.42–0.63	<0.0001	0.50	0.39–0.66	<0.0001	0.50	0.40–0.63	<0.0001
Nadir CD4+ T-cell count	0.76	0.67–0.86	<0.0001	0.85	0.72–1.01	0.0617	0.85	0.73–0.99	0.0329
Zenith viral load	1.00	0.98–1.02	0.8252	1.00	0.98–1.02	0.9687			
Obesity	1.42	0.77–2.63	0.2632	1.06	0.49–2.32	0.8763			

95% CI, 95% confidence interval; TP, therapeutic plan; ^#^ NRTI, NNRTI, PI, II and FI. * Charlson Comorbidity Score (2011 updated version—excluding AIDS); ° scale from 0 to 10.

**Table 3:** Univariate and multivariable multinomial logistic regression with fixed selection predicting a viral blip.

Variables	Multivariable Analysis with Fixed Selection		
	OR	95% CI	*p*-Value
Age, years	1.02	0.99–1.05	0.2783
Duration of therapy, years	0.96	0.92–1.01	0.1108
AIDS	1.97	1.00–3.92	0.0515
Single-tablet regimen for HAART	0.70	0.38–1.29	0.2551
Time since start of current TP, years	0.94	0.84–1.04	0.2071
COVID-19	2.68	1.27–5.68	0.0099
Adherence to therapy °	0.59	0.46–0.75	0.0000
Nadir CD4+ T-cell count	0.84	0.71–1.01	0.0584

95% CI, 95% confidence interval; TP, therapeutic plan; ° scale from 0 to 10.

**Table 4:** Univariate and multivariable logistic regression with fixed selection predicting persistent low-level viremia or virological failure.

Variables	Multivariable Analysis with Fixed Selection		
	OR	95% CI	*p*-Value
Age, years	1.03	0.99–1.06	0.11186
Duration of therapy, years	0.95	0.91–1.00	0.0534
AIDS	1.78	0.85–3.71	0.1259
Single-tablet regimen for HAART	0.50	0.26–0.95	0.0352
Time since start of current TP, years	0.92	0.82–1.03	0.1480
COVID-19	1.79	0.74–4.33	0.1974
Adherence to therapy °	0.42	0.33–0.55	0.0000
Nadir CD4+ T-cell count	0.85	0.70–1.04	0.1107

95% CI, 95% confidence interval; TP, therapeutic plan; ° scale from 0 to 10.

## Data Availability

Data will be provided upon request.
